# Long-term results of a vitamin E–blended polyethylene liner in total hip arthroplasty: a minimum ten year computer aided design based evaluation of wear and component positioning

**DOI:** 10.1007/s00264-026-06997-8

**Published:** 2026-09-04

**Authors:** Eren Demir, Mahfuz Rasuli, Michael Nienhaus, Philipp Schippers, Martin Naisan, Yazan Noufal, Philipp Drees, Yama Afghanyar

**Affiliations:** 1 Department of Orthopaedics and Traumatology, University Medical Center of the Johannes Gutenberg University, Mainz, Germany; 2 Department of Orthopaedics and Traumatology, Johannes Gutenberg University Mainz, Mainz, Germany; 3 Department of Orthopaedic Surgery, St. Josefs Hospital, Wiesbaden, Germany

**Keywords:** Total hip arthroplasty, Vitamin E–blended polyethylene, Polyethylene wear, Acetabular component positioning

## Abstract

**Background:**

Aseptic loosening remains a leading cause of revision in total hip arthroplasty (THA), with polyethylene wear representing a key contributing factor. Vitamin E–blended highly cross-linked polyethylene (VEPE) has been introduced to improve oxidative stability and long-term wear performance; however, long-term clinical data remain limited.

**Methods:**

This retrospective study evaluates long-term polyethylene wear and component positioning in a cohort of patients who underwent primary THA using a vitamin E–blended polyethylene monoblock acetabular component (RM Pressfit vitamys®). Patients were followed for a minimum of ten years. Radiographic assessment was performed using a fully automated, observer-independent CAD-based software (RayMatch®), enabling measurement of cup inclination, anteversion, and polyethylene wear through 2D–3D registration of implant CAD models.

**Results:**

A total of 57 hips with complete radiographic follow-up were analyzed. Mean polyethylene wear rates were 26.9 µm/year at five years and 18.2 µm/year at ten years, remaining well below commonly reported thresholds associated with osteolysis. Cup inclination remained stable over time, whereas a gradual increase in measured anteversion was observed during follow-up.

**Conclusion:**

At a minimum ten year follow-up, the investigated vitamin E–blended polyethylene monoblock cup demonstrated favorable long-term wear characteristics and stable component positioning. Further comparative studies are warranted to compare its long-term performance with other acetabular cup and liner designs.

## Introduction

Total hip arthroplasty (THA) is among the most successful surgical procedures of the last century [1]. Nevertheless, aseptic loosening remains the leading cause of revision surgery, particularly due to polyethylene wear [2]. Over recent decades, several design improvements have been introduced to reduce the risk of aseptic loosening in THA. In addition to changes in implant design, further developments were carried out for the liner. For many years, ultra-high molecular weight polyethylene (UHMWPE) has been considered the gold standard for acetabular liners. A major advancement was the development of cross-linked UHMWPE to improve wear resistance. Highly cross-linked polyethylene (HXLPE), commonly used in modern total hip arthroplasty, is produced by irradiation-induced cross-linking of UHMWPE, which significantly reduces wear while inevitably generating free radicals within the material [3]. Oxidative stress caused by these radicals has been shown to negatively affect the long-term survival of hip implants [4]. In order to eliminate the remaining free radicals and improve oxidative stability, vitamin E (alpha-tocopherol) is incorporated into HXLPE as an antioxidant, contributing to the improvement of mechanical stability [5].

Implant design represents another important factor influencing polyethylene wear. A common distinction is made between monoblock cups and modular metal-backed cups. In monoblock systems, the liner and outer shell are factory-assembled into a single unit, which has been proposed to reduce backside wear [6]. However, the literature remains inconsistent regarding the clinical advantages of monoblock cups [7].

Monoblock cups made of vitamin E–blended highly cross-linked polyethylene (VEPE) are therefore considered to offer high durability and low wear rates compared to other cup designs, although long-term clinical data remain limited [8]. First ten year clinical and radiographic outcomes of VEPE acetabular cups assessed using Einzel-Bild-Röntgen-Analyse (EBRA) demonstrated excellent results, showing low wear rates and prevention of aseptic loosening [9]. However, EBRA is a semi-automatic, graphical method which remains controversial. EBRA evaluates standard anteroposterior radiographs over a period of time. It largely requires high-quality, standardized radiographs and is also observer-dependent. A highly precise method for analyzing micromotion of implants is radiostereometric analysis (RSA); however, traditional RSA requires implantation of tantalum beads, which limits its use in clinical practice [10]. A more recent approach for measuring cup inclination, anteversion, and polyethylene wear is automated analysis using virtual computer-aided design (CAD)–based radiographs**,** which has been validated for polyethylene wear assessment [11, 12].

In this study, we aimed to evaluate the polyethylene wear of a monoblock cup consisting of VEPE over a 10-year period using CAD-based radiographic analysis.

## Methods

This study is a retrospective analysis of long-term polyethylene wear and component orientation in the same cohort of patients previously enrolled in our prospective observational study, in which migration was originally assessed using EBRA and clinical outcomes were recorded [9]. Patients aged 20 to 85 years underwent primary hip arthroplasty between March 2010 and September 2011. Indications for surgery included primary osteoarthritis, secondary osteoarthritis, femoral head necrosis, femoral neck fracture, and congenital dysplasia. In all cases, the RM Pressfit vitamys® cup (Mathys Ltd., Bettlach, Switzerland) was implanted. This uncemented monoblock cup is made of a rough titanium particle surface with a vitamin E-infused highly cross-linked polyethylene (VEPE). Primary stability is achieved by an equatorial press-fit technique, while secondary stability relies on osseous integration of the titanium coating. The acetabular component was combined with various uncemented femoral stems depending on the individual case. All patients received a 28 mm ceramic head. All surgeries were performed via an anterolateral approach with the patient in the supine position.

Patients were followed according to a standardized protocol. Consistent anteroposterior pelvic radiographs were obtained postoperatively, and at one, five and ten years after surgery.

**Fig. 1:**
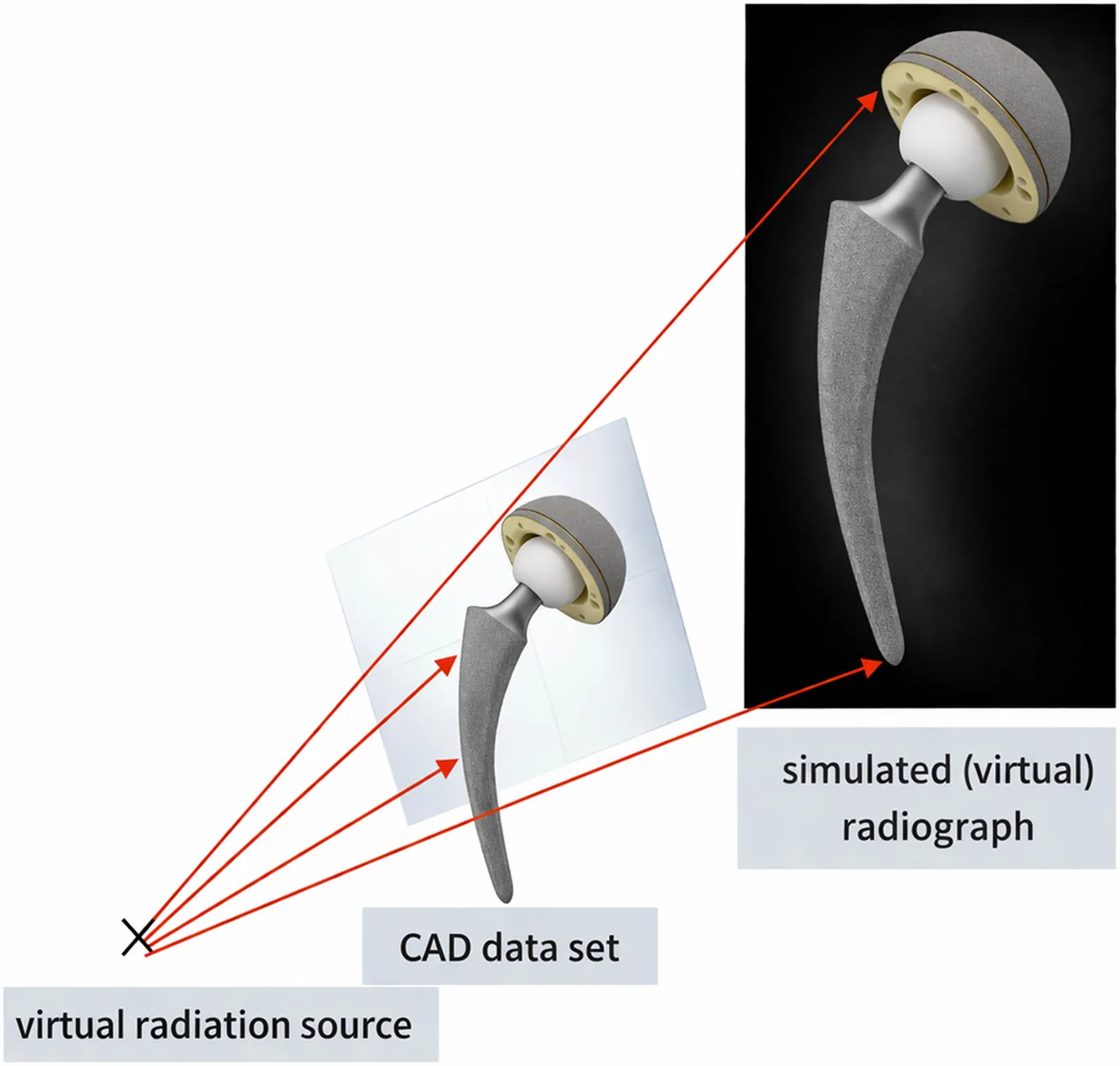
Schematic illustration of the computer-based generation of virtual radiographs. A virtual X-ray source projects the three-dimensional CAD model of the prosthetic components onto a projection plane, producing a simulated radiographic image for further analysis

The radiographs were analyzed using the observer-independent RayMatch® software (Raylytic GmbH, Leipzig, Germany). The underlying automated 2D–3D registration technique has previously been validated for the assessment of polyethylene wear, cup inclination, and cup anteversion [12]. CAD data of the prosthetic components were provided by the manufacturer and were essential for the analysis. The software operates fully automatically and is independent of investigator experience. A virtual reconstruction of the implant components is created using a computer simulation: the CAD models of the cup and femoral head are projected onto a simulated detector from a virtual radiograph source (Fig. 1). Each voxel is assigned material-specific attenuation coefficients, and the radiograph intensity along the projection vectors is calculated using the Lambert–Beer law, producing a realistic digitally reconstructed radiograph (DRR). The DRR is iteratively aligned with the actual radiograph in all six degrees of freedom until the best possible fit is achieved [10].

The following parameters were determined using this method [12]:

- Cup inclination (CupInc): angle between the cup surface and a horizontal reference line, measured via 2D–3D registration of the patient-specific cup CAD model to the radiograph.
- Cup anteversion (CupAnt): angle between the cup surface and its symmetry axis in the frontal plane, determined using the same 2D–3D registration.
- Polyethylene wear: linear distance between the inner cup surface and center of femoral head, calculated in micrometers from 2D–3D registration of the patient-specific cup and head CAD models. The wear rate is expressed as the mean annual value.

Cup inclination values reported in this study correspond directly to the raw measurements provided by the software. The software reports angles in an absolute coordinate system based on the radiograph, which may result in values above 90°. These values were not adjusted and are presented as measured throughout the study to maintain consistency with the software output.

All radiographs were reviewed for standardized image quality and correct pelvic positioning prior to analysis. Radiographs with inadequate pelvic orientation, rotation, or centering were excluded, resulting in different numbers of evaluable radiographs at each follow-up.

Radiographic data and analysis results were obtained using a combination of manual assessments performed by trained and qualified personnel and automated, validated computer-based image analysis routines (FXA image analysis software; FDA 510(k) clearance K110765). All analyses were conducted in accordance with Good Laboratory Practice (GLP) and DIN EN ISO 13485 guidelines. Results are reported descriptively.

All procedures were conducted in accordance with the Declaration of Helsinki (1964). Written and verbal informed consent was obtained from all participants. Ethical approval was granted by the Ethics Commission of the State Medical Association of Hessen, Frankfurt, Germany (approval number: FF 154/2017). The study was registered as an observational trial on clinicaltrials.gov (NCT04322916).

## Results

A total of 96 patients (101 hips) were initially enrolled and followed for a mean duration of 129.3 months (range, 120.0 to 148.9 months). Five patients underwent one-stage bilateral THA. At a minimum follow-up of ten years, complete clinical and radiological data were available for 57 hips (56.4%), which were eligible for CAD-based radiographic analysis using the RayMatch software (Raylytic GmbH, Leipzig, Germany). Seven patients (6.9%) were lost to follow-up, and 23 patients (22.8%) died with the implant in situ. All deaths were unrelated to the surgical procedure and did not occur within the first postoperative year. An additional 14 hips (13.9%) were followed clinically but did not undergo radiographic evaluation at long-term follow-up, primarily because patients declined further radiographs in the absence of hip-related symptoms.

Among the 57 hips with available radiographic data, not all radiographs were evaluable at every follow-up time point due to insufficient image quality or inadequate pelvic positioning. Consequently, the number of radiographs available for CAD-based analysis varied between individual follow-up intervals. Although radiographs were obtained at additional time points as part of the original prospective follow-up protocol, the present RayMatch-based analysis was restricted to predefined follow-up intervals (postoperative, 1 year, 5 years and 10 years), and only radiographs corresponding to these selected time points were included.

The most common indication for surgery was primary osteoarthritis (93%), followed by femoral head necrosis (3%), secondary osteoarthritis (2%), femoral neck fracture (1%), and congenital dysplasia (1%). Baseline demographic characteristics and diagnoses of the initial cohort are summarized in Table 1.

**Table 1:** Baseline characteristics, follow-up status, and primary diagnoses of the study cohort

Variable	Female	Male	Total
Hips, n	63	38	101
Mean age, yrs (range)	70.3 (50.7–84.0)	67.9 (51.0–84.3)	69.4 (50.7–84.3)
Mean BMI, kg/m^2^ (range)	27.3 (19.3–39.1)	27.9 (20.2–41.5)	27.5 (19.3–41.5)
Follow-up Status, n			
Hips with complete clinical and radiological follow-up (≥ 10 years)			57
Hips with clinical follow-up only (no radiographs at ≥ 10 years)			14
Patients lost to follow-up			7
Patients who died with the implant in situ			23
Primary diagnosis, n (%)			
Primary osteoarthritis			94
Secondary osteoarthritis			2
Femoral head necrosis			3
Femoral neck fracture			1
Congenital dysplasia			1

**Fig. 2:**
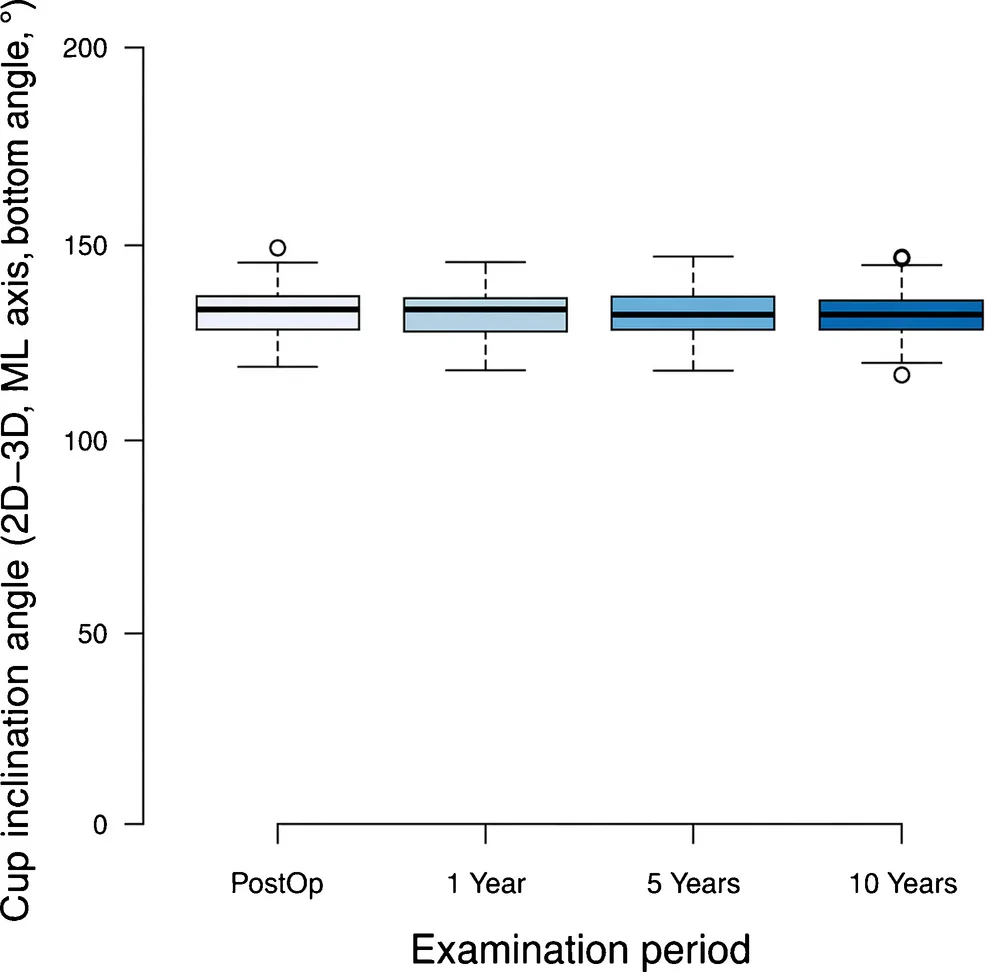
Boxplots showing acetabular cup inclination measured immediately postoperatively and at 1, 5, and 10 years after surgery. The boxes represent the interquartile range with the median indicated, whiskers denote the range, and circles indicate outliers

Cup inclination measurements obtained from the CAD-based software are summarized in Table 2. Postoperative measurements were available for 46 hips, with a mean inclination of 133.5° (median 133.7°, standard deviation [SD] 5.4°, range 120.2°–147.9°). At 1 year, 39 hips were assessed, showing a mean inclination of 132.3°. At five years, 54 hips demonstrated a mean inclination of 132.9°, and at ten years, 51 hips were evaluated with a mean inclination of 133.0° (Fig. 2).

**Table 2:** Descriptive statistics of acetabular cup inclination (degrees) measured immediately postoperatively (PostOp) and at 1, 5, and 10 years after surgery. Data are reported as mean, median, standard deviation (SD), maximum (Max), and minimum (Min)

Examination period	Measurements	Mean	Median	SD	Max	Min
PostOp	46	133.5	133.7	5.4	147.9	120.2
1 Year	39	132.3	133.7	6.0	143.9	118.9
5 Year	54	132.9	132.3	6.0	145.9	118.8
10 Year	51	133.0	132.4	5.9	146.2	117.9

**Fig. 3:**
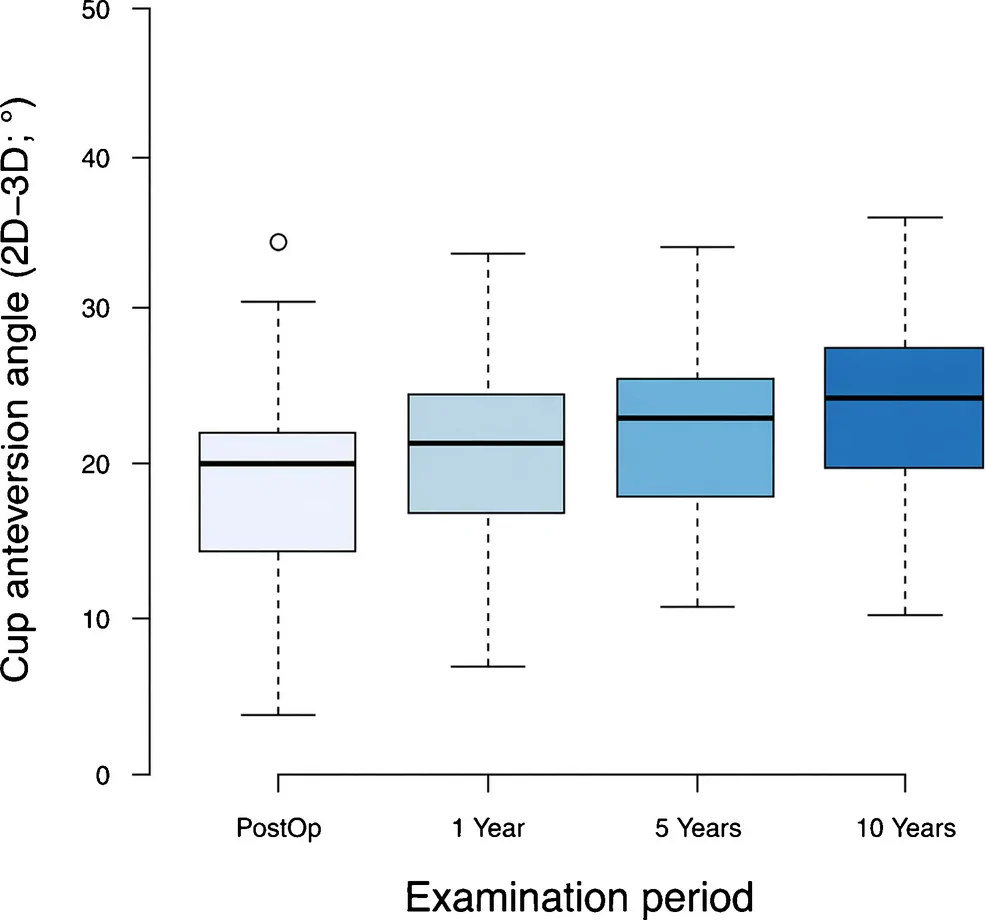
Boxplots showing acetabular cup anteversion measured immediately postoperatively and at 1, 5, and 10 years after surgery. The boxes represent the interquartile range with the median indicated, whiskers denote the range, and circles indicate outliers

Cup anteversion measurements are presented in Table 3. Postoperative data for 46 hips showed a mean anteversion of 18.7°. At one year, 39 hips had a mean anteversion of 20.9°. At five years, 54 hips demonstrated a mean anteversion of 22.2°, and at ten years, 51 hips were assessed with a mean anteversion of 23.6° (Fig. 3).

**Table 3:** Descriptive statistics of acetabular cup anteversion measured immediately postoperatively (PostOp) and at 1, 5, and 10 years after surgery. Data are reported as mean, median, standard deviation (SD), maximum (Max), and minimum (Min)

Examination period	Measurements	Mean	Median	SD	Max	Min
PostOp	46	18.7	20.2	6.0	33.8	4.0
1 Year	39	20.9	21.3	5.8	33.3	7.1
5 Year	54	22.2	22.9	5.4	33.6	11.1
10 Year	51	23.6	24.2	5.8	35.8	10.5

**Fig. 4:**
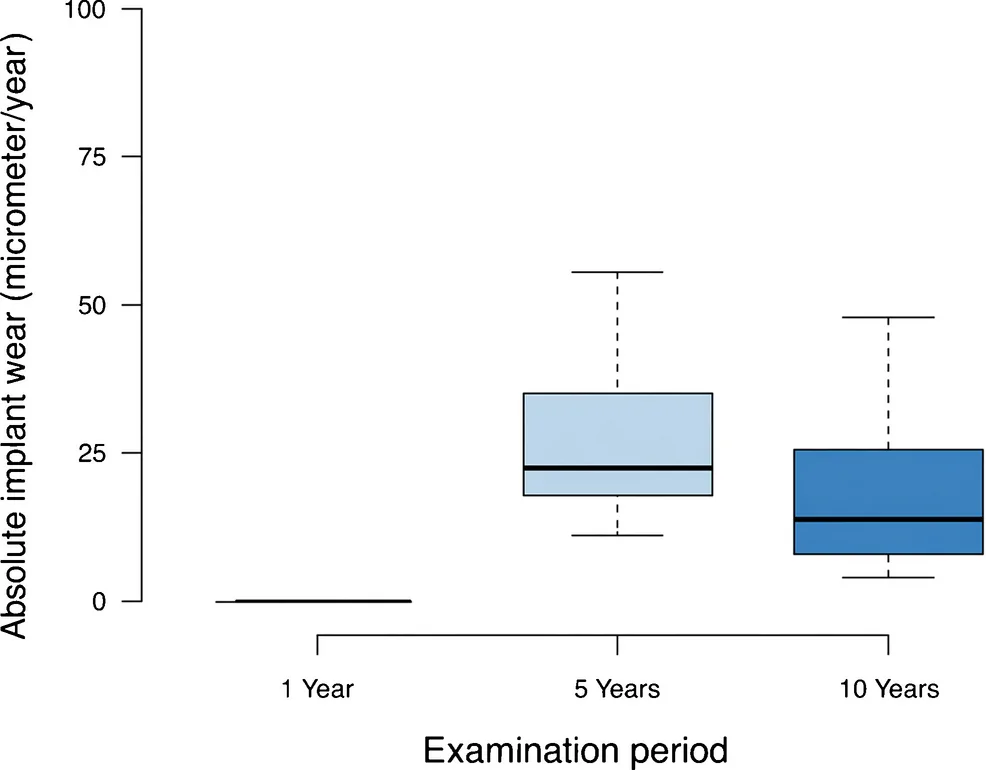
Boxplots showing absolute polyethylene wear expressed as micrometers per year at 1, 5, and 10 years after surgery. The boxes represent the interquartile range with the median indicated, whiskers denote the range, and circles indicate outliers

Polyethylene wear results are summarized in Table 4. Wear was calculated from the change in the spatial relationship between the femoral head centre and the inner surface of the polyethylene liner at each follow-up time point relative to the one year follow-up, using 2D–3D registration of patient-specific cup and head CAD models to the radiograph. Values are reported in micrometers and expressed as the average annual wear rate. At five years, 39 hips demonstrated a mean wear rate of 26.9 µm/year, and at ten years, 37 hips were assessed with a mean wear rate of 18.2 µm/year (Fig. 4).

**Table 4:** Descriptive statistics of polyethylene wear (µm) measured at 1, 5, and 10 years after surgery. Data are reported as mean, median, standard deviation (SD), maximum (Max), and minimum (Min)

Examination period	Measurements	Mean	Median	SD	Max	Min
1 Year	39	0.0	0.0	0.0	0.0	0.0
5 Year	39	26.9	23.2	10.8	54.2	8.8
10 Year	37	18.2	15.3	10.2	48.9	4.7

## Discussion

In this study, we evaluated long-term radiographic outcomes of a vitamin E–stabilized polyethylene acetabular component using CAD-based analysis in a cohort with a minimum follow-up of ten years. Polyethylene wear remained consistently low over time, with mean annual wear rates of 26.9 µm/year at five years and 18.2 µm/year at ten years. These findings indicate excellent long-term wear performance of the vitamin E–stabilized polyethylene liner and stable acetabular component positioning.

The question of the optimal acetabular component, and particularly the most suitable liner in THA, remains a subject of controversial debate [13]. The RM Pressfit vitamys cup has demonstrated excellent survival rates and low wear rates in both mid- and long-term data [9, 13, 14]. The potential benefits of adding vitamin E to polyethylene liners compared with highly cross-linked polyethylene remain controversial. In a randomized controlled trial with a five-year follow-up, Rochcongar et al. demonstrated reduced wear of the RM Pressfit vitamys cup, indicating superior performance of VEPE compared with a UHMWPE cup. The measurements were performed using radiostereometric analysis (RSA) [15]. Galea et al., in contrast, observed in both liner types similar bedding-in and low wear during the first five postoperative years, with VEPE being independently associated with lower overall wear and a reduced wear rate between the second and fifth year, likely due to its higher crosslinking density from radiation treatment [16]. The measurements were also performed using RSA. Complementing these findings, a recent meta-analysis by Li et al. synthesized data from nine randomized controlled trials and reported that VEPE liners showed significantly reduced vertical and total femoral head penetration, as well as lower steady-state wear rates in early and mid-term follow-up. However, across the included trials, the control groups consisted of either conventional UHMWPE or HXLPE. This heterogeneity in comparator materials may have influenced the pooled effect estimates. Moreover, Li et al. also noted increased cup migration at two years and no clear clinical advantage in mid-term functional outcomes or revision rates, emphasizing the need for longer-term RCTs to clarify the true clinical relevance of VEPE [17].

To our knowledge, the only CAD-based study reported no differences in wear behavior following the addition of vitamin E to highly cross-linked polyethylene liners at five-year follow-up. Interestingly, in this cohort, cup anteversion increased by nearly 5° during the first year after implantation, which the authors attributed to functional adaptation [10].

The methodology for measuring polyethylene wear remains heterogeneous, and no universally accepted thresholds for clinically relevant wear have been established. Reported thresholds are largely derived from specific measurement techniques, such as EBRA or RSA. However, a wear rate of less than 0.1 mm/year (100 µm/year) is rarely associated with osteolysis, and a practical threshold of 0.05 mm/year (50 µm/year) has been suggested to effectively prevent osteolysis [18]. Other authors have proposed a higher “critical threshold” of 0.2 mm/year (200 µm/year) for the development of osteolysis, highlighting the potential utility of linear wear measurements in assessing implant longevity [19]. Overall, a continuous dose–response relationship has been reported, in which the risk of osteolysis rises progressively with increasing polyethylene wear rather than being associated with a discrete critical value [20]. More recently, Elke and Rieker confirmed that wear rates under 0.1 mm/year correlated with osteolysis-free survival of up to 20 years across various femoral head sizes, supporting the concept that maintaining low wear rates is critical for long-term implant longevity [21].

In our data at five years, 39 hips demonstrated a mean wear rate of 26.9 µm/year and at ten years 37 hips demonstrated a mean wear rate of 18.2 µm/year. These results are well below the established thresholds for polyethylene wear and the associated risk of osteolysis, which indicates an exceptionally low wear rate of the VEPE liner.

Throughout the entire follow-up period, no meaningful change in cup inclination was observed. The median inclination measured immediately postoperatively (133.5°) remained virtually unchanged at ten years (133°). This finding supports the presumed primary stability achieved through the press-fit technique as well as the long-term secondary stability provided by reliable osseointegration of the titanium-coated cup. Interestingly, we observed a progressive increase in acetabular anteversion over time. The mean anteversion measured was 18.7° immediately postoperatively, 20.9° at one year, 22.2° at five years, and 23.6° at ten years. A similar finding has been reported in a previous study using a CAD-based radiographic analysis. The authors attributed this phenomenon not to true rotational migration of the cup—since permanent and stable osseointegration of titanium cups typically occurs early after implantation—but rather to functional and radiographic factors. Variations in pelvic tilt over time can substantially influence the measured anteversion in two-dimensional radiographs, whereas inclination remains largely unaffected. Because CAD-based automated measurement techniques, including the one used in our study, do not compensate for changes in pelvic tilt, these positional variations may present as an apparent increase in anteversion [10]. We share the authors’ interpretation that the progressive rise in measured anteversion likely reflects pelvic positional effects rather than true implant rotation.

A major strength of this study is the use of investigator-independent, CAD-based radiographic analysis. To date, this is the first study providing long-term results using this methodology, as previously published CAD-based studies were limited to shorter follow-up periods. The only available long-term data on vitamin E–infused polyethylene liners were obtained using EBRA. However, EBRA is associated with a relatively high dropout rate due to strict radiographic quality requirements. CAD-based radiographic analysis similarly depends on adequate image quality and correct pelvic positioning, requiring exclusion of radiographs that do not meet predefined criteria. Nevertheless, its automated and investigator-independent nature minimizes observer-related variability and provides a robust alternative for long-term polyethylene wear assessment.

Radiostereometric analysis (RSA) is widely regarded as the most precise method for assessing implant micromotion and wear; however, its implementation is relatively complex, limiting its use in clinical practice to a small number of patients [22]. The CAD-based software has been validated for wear measurements and therefore represents a reliable alternative to RSA, providing a feasible method for long-term implant monitoring in larger patient cohorts [11]. The CAD-based radiographic analysis used in the present study has previously been validated by Klebingat et al. for the accurate determination of polyethylene wear, cup inclination, and cup anteversion using automated 2D–3D registration [12]. Accordingly, the aim of the present study was not to validate the measurement technique itself or compare it with conventional radiographic methods, but rather to apply this previously validated methodology for long-term assessment of implant performance.

Our study design nevertheless presents several limitations. First, the relatively small patient cohort in this single-center study must be acknowledged. Additionally, some follow-up data could not be utilized due to limitations in radiographic image accuracy, resulting in further exclusions. Also, some patients refused radiographic follow-up examinations because they were asymptomatic and satisfied with their hip function, leading to incomplete imaging data. Therefore, the present study should be interpreted in the context of its observational single-cohort design. As no control group including conventional HXLPE liners or alternative acetabular cup designs was available, direct comparisons cannot be made. Consequently, our findings demonstrate favourable long-term wear characteristics of the investigated implant but do not allow conclusions regarding its superiority over other polyethylene materials or cup concepts.

A potential limitation of the CAD-based software appears to be the measurement of cup anteversion. However, as observed over the years and similarly reported in previous studies, the anteversion increases without indicating actual cup loosening and should, as described above, be interpreted as a functional phenomenon. Another limitation of our study is that the CAD-based software, while validated for wear measurement, does not provide precise data on cup migration, a crucial parameter for predicting long-term implant loosening. Early acetabular cup migration has been shown to correlate significantly with future aseptic loosening. For instance, a systematic review and meta-analysis of RSA studies reported that 36% of cohorts exhibited proximal migration greater than 0.2 mm at two years—a threshold previously associated with an increased risk of loosening [23].

Furthermore, the inclusion of different femoral stem designs represents a potential confounding factor, although the primary focus of this study was on the acetabular component and liner. Additionally, it would be of interest to investigate potential differences between modular and monoblock acetabular cups, as the design may influence long-term wear and implant stability. Several studies have addressed the debate between modular and monoblock acetabular cups. A matched-pair study compared a VEPE monoblock cup with a modular metal-back cup using HXLPE without an antioxidant and found no significant differences in mid-term cup migration or polyethylene wear, as assessed using EBRA, while implant survival was 100% in both groups [8]. Another matched-pair prospective randomized trial found that an isoelastic monoblock cup (RM Pressfit vitamys) resulted in significantly less proximal bone loss compared with a modular metal-back cup after four years, especially in the polar acetabular region [24]. A systematic review found no convincing evidence that monoblock components have lower polyethylene wear, migration, osteolysis, or aseptic loosening compared with modular designs at short- to intermediate-term follow-up (approximately 2 to 8 years) [7]. Moreover, a comparative study with non-modular porous-coated cups showed significantly lower rates of osteolysis and more consistent wear compared with modular components, likely due to reduced micromotion and better liner-shell conformity, at intermediate-term follow-up of approximately five years (mean follow-up, 5.3 to 5.5 years) [6]. These findings emphasize that a systematic comparison of monoblock versus modular designs in the context of vitamin E–infused liners could be highly relevant.

Moreover, while our study provides ten-year follow-up data, which is considered relatively long compared with existing literature, even longer-term data are needed to fully evaluate the durability and performance of VEPE liners.

Multicenter studies with larger patient cohorts are necessary to confirm the generalizability of our findings and to better assess the long-term performance of vitamin E–infused polyethylene liners across diverse surgical techniques and patient populations. In this context, a prospective multicenter observational study using EBRA demonstrated that femoral head size did not influence mid-term polyethylene wear, cup migration, or clinical outcomes of vitamin E–infused highly cross-linked polyethylene acetabular cups at a mean follow-up of approximately four to five years [25]. However, longer-term follow-up is required to determine whether these favourable mid-term results translate into sustained long-term implant performance.

## Conclusion

In conclusion, our ten-year follow-up demonstrates that the RM Pressfit vitamys cup with a vitamin E–infused polyethylene liner shows excellent long-term stability, minimal wear, and a very low risk of osteolysis. The observed increase in anteversion over time appears to reflect functional and radiographic factors rather than true implant migration. Larger and comparative multicenter studies are warranted to confirm these findings and to further evaluate the long-term performance of this implant relative to other acetabular cup and liner designs.

## Data Availability

The datasets generated and/or analyzed during the current study are available from the corresponding author on reasonable request.
